# A Case Series of Completely Thrombosed Abdominal Aortic Aneurysms

**DOI:** 10.3390/jcdd12030098

**Published:** 2025-03-11

**Authors:** Raffaello Bellosta, Giulia Anna Sala, Marco Franchin, Luca Luzzani, Alessandro Pucci, Gabriele Piffaretti, Maria Cristina Cervarolo

**Affiliations:** 1 Vascular Surgery—Poliambulanza Hospital, Via Bissolati 57, 25124 Brescia, Italy; raffaello.bellosta@poliambulanza.it (R.B.); luca.luzzani@poliambulanza.it (L.L.); alessandro.pucci@poliambulanza.it (A.P.); 2 Vascular Surgery—Department of Medicine and Surgery, University of Insubria School of Medicine, Via Guicciardini, 9, 21100 Varese, Italy; gasala@studenti.uninsubria.it (G.A.S.); marco.franchin@asst-settelaghi.it (M.F.); mariacristina.cervarolo@asst-settelaghi.it (M.C.C.); 3 Department of Cardio-Thoracic and Vascular Surgery, ASST Settelaghi University Teaching Hospital, Via Guicciardini, 9, 21100 Varese, Italy

**Keywords:** thrombosed abdominal aortic aneurysm, acute thrombosis, abdominal aortic aneurysm

## Abstract

Background: Completely thrombosed AAA (th-AAA) has been infrequently described in the literature. The present study evaluated the incidence and report the outcomes of open surgical repair (OSR) of a clinical series of th-AAAs. Methods: This is a single-center, observational cohort study of consecutive th-AAAs identified between 10 October 1998, and 31 January 2024. Open repair was carried out through a transperitoneal route, and Dacron knitted graft replacement. Follow-up included the clinical visit and duplex ultrasound at 30 days, and annually thereafter. The primary outcome was overall survival. The secondary outcome was the freedom from aorta-related reintervention. Results: Out of 2237 AAA repairs, we identified 16 (0.7%) th-AAAs. They were all men with a mean age of 74 years ± 8 (range, 54–89). The median of aneurysm diameter was 49 mm (IQR, 46–52). Rupture was the presenting scenario in four (25%) patients. Early mortality and major amputation did not occur. At a mean follow-up of 70 months ± 48 (range, 11–192), the freedom from aorta-related mortality was 100%, and graft-related complications were not observed. Conclusions: The incidence of th-AAA was <1%. Although rupture was the presenting scenario in nearly 25% of the cases, OSR was safe and effective due to the absence of aorta-related mortality and the long-term durability of the repair.

## 1. Introduction

The widespread use of screening programs through ultrasound as well as the extensive use of computed tomography angiography in different clinical scenarios hasimproved the detection of abdominal aortic aneurysms (AAAs) [1]. However, completely thrombosed AAA (th-AAA) has been described rarely in the literature; the reported incidence ranged from 0.6%to2.8% of all surgically managed AAAs [2,3]. Robust clinical series have not been published so far, and cardiovascular guidelines do not include specific recommendations to define the standard of care for these challenging lesions [4,5]. The present study evaluated the incidence and report the outcomes of open surgical repair (OSR) of a clinical series of th-AAAs.

## 2. Materials and Methods

**Figure 1:**
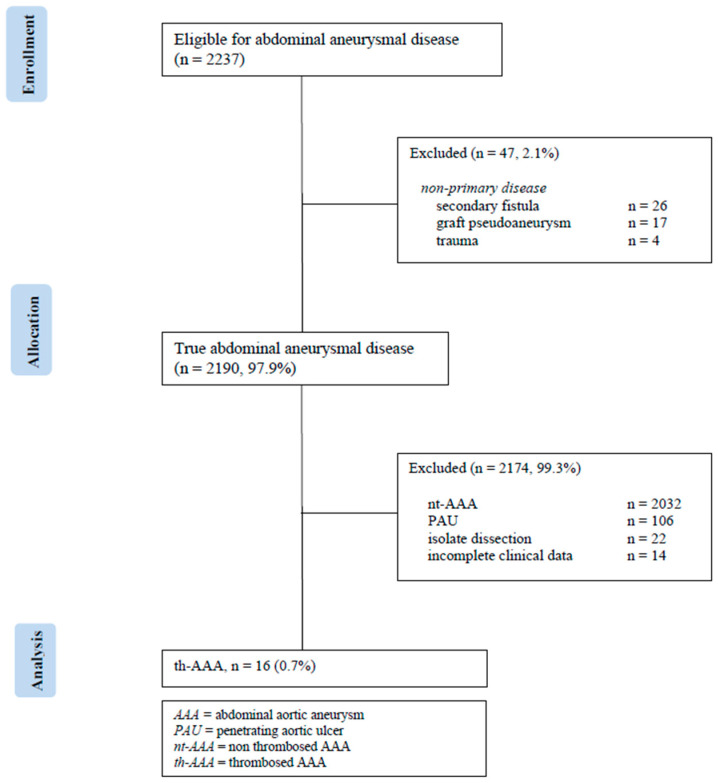
Consort diagram of open surgical aortic repair for thrombosed abdominal aortic aneurysm (1998 to 2024, n = 16).

Consent for publication of anonymized data was waived by the local Institutional Review Board (n.151/2024) [6]. This is a single-center, observational cohort study of consecutive th-AAAs identified between 10 October 1998 and 31 January 2024 (Figure 1).

Clinical data were analyzed retrospectively.

### 2.1. Definitions

**Figure 2:**
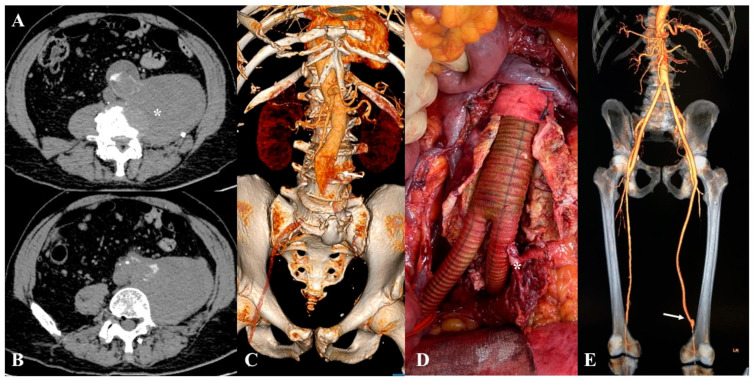
Preoperative computed tomography angiography (**A**,**B**) showing the infrarenal abdominal aortic aneurysm rupture into the left psoas muscle (asterisk). Volume rendering 3D reconstruction highlighted the site of rupture ((**C**), white arrow). Intraoperative finding (**D**) the final reconstruction after aortic graft replacement using a bifurcated graft with the inferior mesenteric artery transposition (asterisk). Follow-up computed tomography angiography at 48 months follow-up confirmed the absence of graft-related complication and the patency of the adjunctive above-the-knee femoro-popliteal reconstruction ((**E**), white arrow).

A th-AAA was defined as an aneurysm with a completely occluded lumen (Figure 2A–C).

All grading systems and operative outcomes were defined according to the Society for Vascular Surgery (SVS) practice guidelines on the care of patients with an AAA [5]. The follow-up index (FUI) describes the completeness of follow-up at a given study end date and the ratio of the period investigated to the potential follow-up period. For this specific study, the primary outcome was overall survival. The secondary outcome was the assessment of freedom from aorta-related reintervention.

### 2.2. Technical Details

Preoperative antibiotic prophylaxis was performed with a second-generation cephalosporin in all cases. Heparinization was administered at a dose of 40 UI/kg, eventually adjusted in correlation with the estimated glomerular filtration rate in patients with chronic kidney disease. Open repair was carried out through a transperitoneal route; the site of aortic cross-clamping as well as the type of graft replacement was determined by proximal extent of the aortic lesion and iliac artery involvement, respectively. Throughout the entire experience, we used only Dacron knitted graft (Intergard Silver^®^/Synergy^®^—Getinge; Gothenburg, Sweden) for the aortic reconstruction (Figure 2D,E). Inferior mesenteric artery (IMA) transposition was left at surgeon’s discretion, but performed when it measured >3 mm in diameter, or in the presence of active back bleeding [7]. All patients were admitted in the intensive care unit postoperatively. Laboratory analyses always included blood count, coagulation, serum lactic acid concentration, and a full visceral enzymes panel daily. Follow-up included a clinical visit and duplex ultrasound at 30 days, and annually thereafter.

### 2.3. Statistical Analysis [8]

Statistical analysis was performed with SPSS, release 29.0 for Windows (IBM SPSS Inc.; Chicago, IL, USA). Continuous variables were tested for normality using the Shapiro–Wilk test, and compared between groups with an unpaired Student’s *t*-test for normally distributed values; otherwise, the Mann–Whitney U test was used. Variables that were normally distributed are presented as mean ± standard deviation and range; otherwise, they are presented as median and interquartile range (IQR). Categorical variables were presented using frequencies and percentages and analyzed with Fisher’s exact test. Long-term survival and freedom from aorta-related reintervention were estimated according to Kaplan–Meier method and reported with standard error (SE) and associated 95% confidence intervals (95%CI). All reported P values were two-sided; *p* value <0.05 was considered significant.

## 3. Results

### 3.1. Study Population

We identified 16 (0.7%) cases out of the entire aortic cohort. They were all men with a mean age of 74 years ± 8 (range, 54–89). The median of aneurysm diameter was 49 mm (IQR, 46–52): aneurysm extent was infrarenal in 14 (87.5%), and juxtarenal in two (12.5%). One juxtarenal aneurysm was inflammatory in etiology. There were neither systemic nor local signs of infection. Aortic thrombosis extended to the iliac-femoral arteries bilaterally in all but two (87.5%) cases. Demographic data risk factors and clinical presentation are reported in Table 1.

Overall, all th-AAAs were clinically symptomatic. Briefly, four (25%) patients had ruptured th-AAAs (contained, n = 3; free, n = 1); all ruptures developed from the thrombosed aneurysmatic sac, with the free rupture that developed into the left psoas muscle (Figure 2A,B). Clinically, all non-ruptured th-AAAs were symptomatic: nine (56%) were diagnosed because the patient presented with chronic limb threatening ischemia, whereas in five (31.5%) cases the AAA was acutely thrombosed leading to acute limb ischemia. In two (12.5%) patients, the th-AAA was ruptured and associated with acute limb ischemia.

### 3.2. Procedural Details

Urgent intervention was performed in seven (44%) patients. We performed aneurysm graft replacement with an aorto-bifemoral bypass in 15 (94%) patients, and with an aorto-bi-iliac interposition graft in one (6%). Intraoperatively, eight (50%) patients required one or more additional procedures (Table 2).

The median of operation time was 220 min (IQR, 190–242.5), and was higher in patients requiring additional procedures [minutes, 241 (IQR, 235.2–288) vs. 190 (IQR, 185–194.5), *p* = 0.001). Overall, the median estimated blood loss was 500 mL (range, 500–1170), but it was not different in case of urgent surgery (*p* = 0.315) or need of an additional procedure (*p* = 0.615).

### 3.3. Postoperative Outcomes

In-hospital mortality did not occur. Four (25%) patients developed a major postoperative complication: acute kidney injury (n = 1), pneumonia (n = 1), groin hematoma (n = 1), and ventral hernia (n = 1) that required relaparotomy. Ischemic-reperfusion syndrome developed in three (19%) patients; major amputation was never required in these patients. The median length of hospitalization was 7 days (IQR, 7–12), with no difference between urgent and elective operations (*p* = 0.302). The FUI was 1: at a mean follow-up of 70 months ± 48 (range, 11–192), eight (50%) patients died. Estimated survival was 88% (SE: 0.8) at 36 months; the freedom from aorta-related mortality was 100%. At the last radiologic follow-up available, no graft-related complications were observed.

## 4. Discussion

**Figure 3:**
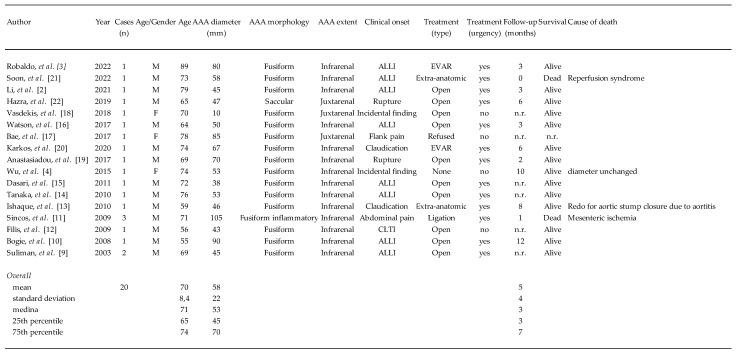
Summary of the recent literature dealing with thrombosed abdominal aortic aneurysm. (n, number; M, Male; F, Female; AAA, abdominal aortic aneurysm; ALLI, acute lower limb ischemia; CLTI, critical limb threatening ischemia; n.r., not reported; EVAR; endovascular aortic repair) [2,3,4,9,10,11,12,13,14,15,16,17,18,19,20,21,22].

Our series confirms that th-AAA is a rare finding within an aortic aneurysm cohort; the incidence of 0.7% is in line with what previously reported in the literature (Figure 3) [2,3,4,9,10,11,12,13,14,15,16,17,18,19,20,21,22].

However, the most important aspect is that, unlike previous publications based on single clinical cases or very small (≤3 cases) series, our data are based on a more robust case series. Whether uncertainty regarding the true incidence of th-AAA may derive from different reasons such as different definitions and reporting criteria or because it is simply underreported is yet to be fully demonstrated.

Reviewing the most recent literature, we found 20 th-AAAs overall; the literature data are significantly different in comparison with our findings, especially from a clinical point of view: rupture was an infrequent finding at 5%, while it was 25% in our group and it was independent of the diameter of the th-AAA since three aneurysms ruptured for diameter < 55 mm [11,17,19]. Our data find some support in the paper of Robaldo et al. [3] who underlined that there is no definitive relationship between aneurysm size and the likelihood of thrombosis or rupture of th-AAA. It is noteworthy to underline that, acutely th-AAA carried a mortality rate as high as 53% in the literature, similar to that of non-thrombosed ruptured AAA [4]. Endovascular aortic repair has been advocated as an appealing alternative even in these demanding lesions, but the results of OSR in our hands were safe and effective with no early mortality and no aorta-related mortality during the follow-up [3].

Among different technical strategies, endovascular repair may be at increased risk of complication due to thrombus dislodgment, especially in case of juxtarenal extent of the th-AAA. Aortic ligation with extra-anatomical reconstruction through an axillo-bifemoral bypass has been advocated to relieve rapidly the ischemic injury of the lower limbs [13,21]. We did not appreciate this type of solution that, at least at our institution, has been reserved only for selected patients deemed unfit due to prohibitive risk for aortic cross-clamp or in selected cases of infected aortic graft. In fact, in the literature it has been reported that the th-AAA remains at risk of rupture due to expansion caused by some sort of endotension [4]. This is the main reason why, by default, we always preferred to proceed with a standard prosthetic graft.

## 5. Conclusions

The incidence of th-AAA was <1%. Despite rupture was the presenting scenario in 25% of the cases, OSR was safe and effective due to the absence of aorta-related mortality and the long-term durability of the repair.

## Figures and Tables

**Table 1:** Demographic data, comorbidities, and risk factors of the cohort.

Covariate	Patients
	(total, n = 16)
Demographics	
Male, n (%)	16 (100)
Age, mean (SD)	74 (8)
>80-year	4 (25.0)
Comorbidities, n (%)	
Hypertension	16 (100)
Smoking habit	14 (87.5)
Dyslipidemia	14 (87.5)
Chronic obstructive pulmonary disease	7 (44)
Coronary artery disease	2 (12.5)
Diabetes	2 (12.5)
Cancer	2 (12.5)
Symptoms, n (%)	
Abdominal pain	4 (25.0)
Hemorrhagic shock	2 (12.5)
Acute lower limb ischemia	5 (31.5)
Chronic limb ischemia	9 (56)

n, number; SD, standard deviation.

**Table 2:** Intraoperative additional procedures.

Covariate	Patients
	(total, n = 16)
Additional procedure, n (%)	13
IMA transposition	4 (25)
Femoral endarterectomy/patch	3 (19)
Femoro-popliteal thrombectomy	3 (19)
Juxtarenal aortic endarterectomy	1 (6)
Hypogastric artery bypass	1 (6)
Femoro-popliteal bypass	1 (6)

n, number; IMA, inferior mesenteric artery.

## Data Availability

Data entry was managed by physicians involved in patient care at each center, then merged into a shared single dataset by the corresponding author (G.Pi.). The data underlying this article will be shared on reasonable request to the corresponding author.
